# The in vitro and in vivo wound-healing effects of royal jelly derived from *Apis mellifera* L. during blossom seasons of *Castanea mollissima* Bl. and *Brassica napus* L. in South China exhibited distinct patterns

**DOI:** 10.1186/s12906-020-03138-5

**Published:** 2020-11-23

**Authors:** Yan Lin, Meng Zhang, Luying Wang, Tianxing Lin, Guanggao Wang, Jianhua Peng, Songkun Su

**Affiliations:** 1 College of Animal Sciences (College of Bee Science), Fujian Agriculture and Forestry University, 15 Shangxiadian Road, Cangshan District, Fuzhou, 350002 China; 2 Apicultural Research Institute of Jiangxi Province, Nanchang, 330052 China

**Keywords:** Royal jelly, Wound healing, Extracts, Proliferation, Migration, Anti-inflammation

## Abstract

**Background:**

Non-healing wounds have been a severe issue in the global healthcare system. Regrettably, royal jelly, a traditional remedy for various skin injuries, has not been widely applied in cutaneous wounds in clinical practice nowadays, which may be due to the confusion and the lack of knowledge about the efficacies of different types of royal jelly, the bioactive constituents, and the precise mechanisms underlying the wound repairing activity. Since the compositions and bioactivities of royal jelly are predominantly influenced by nectar plants, this study aims to explore the differences in the wound-healing properties of royal jelly produced by *Apis mellifera* L. during the blossom seasons of different floral sources, to provide guidelines for the future rational application of royal jelly in cutaneous wounds, and to promote the further discovery of wound repair-promoting substances.

**Methods:**

Royal jelly samples were harvested during flowering seasons of *Castanea mollissima* Bl. (chestnut) and *Brassica napus* L. (rapeseed) in South China, from which hydrophilic and lipophilic fractions were extracted. The in vivo wound-healing potential was preliminarily assessed in Wistar rats’ excisional full-thickness wounds, followed by investigating the mechanisms of action through in vitro assays with human epidermal keratinocytes and LPS-stimulated inflammation in macrophages.

**Results:**

The results indicated that different royal jelly samples exhibited distinct wound-healing potential, in which *Castanea mollissima* Bl. royal jelly was more potent. It sped up wound closure between day 2 and day 4 to 0.25 cm^2^/day (*p* < 0.05), and could accelerate wound repair by enhancing the proliferative and migratory capabilities of keratinocytes by 50.9% (*p* < 0.001) and 14.9% (*p* < 0.001), modulating inflammation through inhibiting nitric oxide production by 46.2% (*p* < 0.001), and promoting cell growth through increasing the secretion of transforming growth factor-β by 44.7% (*p* < 0.001). In contrast, *Brassica napus* L. royal jelly could regulate inflammation by reducing the amount of tumour necrosis factor-α by 21.3% (*p* < 0.001).

**Conclusions:**

The present study improves the application of royal jelly for curing difficult-to-heal wounds, in which the hydrosoluble extract of *Castanea mollissima* Bl. royal jelly promises the greatest potential. It also provides clues which may lead towards the identification of substances derived from royal jelly to treat wounds.

## Background

Bee products, produced by honeybees and maintained in bee hives, including honey, royal jelly, propolis, pollen, venom and bee bread, have been important supplementary medicines since ancient times, due to their broad pharmacological efficacies. Amongst these natural products, royal jelly is known as a “superfood”, due to its powerful potency for queen-differentiation of honeybees and the longevity of the queen bee, as well as its nutritional and therapeutic values for human beings [1, 2]. It is an extraordinarily diverse warehouse of chemical compositions, mainly composed of water, proteins, carbohydrates, lipids, vitamins, free amino acids and minerals [2, 3], and exerting a wide range of biological activities involving antimicrobial [4], anti-inflammatory [5], antioxidant [6], immunomodulatory [7], anti-tumour [8], antidiabetic [9] and wound-healing [10–12] effects. These health-promoting activities make royal jelly a potential therapeutic supplement for diseases such as inflammation, cancer, diabetes, hypertension and wounds [3]. Although possessing various bioactivities, over the past decades, extensive studies tend to concentrate on the immunomodulatory and antibacterial functions of royal jelly, and the mechanisms of some traditional applications such as wound repair, are rarely studied. Previous studies reported that topical application of royal jelly was effective against wounds with or without infection, as well as diabetic foot ulcers [10–12]. Yet, whether or not different kinds of royal jelly possess distinct potencies, and what the bioactive components and mechanisms underlying such efficacy are, are still largely unknown.

In normal physiological conditions, wound healing can complete naturally in a sophisticated manner, within a certain period of time, in which predominant cells (keratinocytes, fibroblasts and macrophages), cytokines (tumour necrosis factor-α, interleukin-1β, interleukin-6, and transforming growth factor-β1), and mediators (nitric oxide and nitric oxide synthase) play critical roles [13–15]. More specifically, proliferation and differentiation of fibroblasts promote the constitution of granulation tissue and connection of gaping wound edges; growth and migration of keratinocytes facilitate re-epithelialisation of wounds, protecting them from infection and desiccation; even though macrophages do not directly participate in the filling of defect, they have phagocytic effects on bacteria and necrotic tissue, and are responsible for regulating the wound healing process by secreting a variety of regulators [14, 16]. Any interruption in the natural healing cascade may lead to non-healing wounds, chronic wounds or scarring [14]. In reality, the prevalence of chronic wounds has become worse in recent years owing to population aging and the rise in the incidence of diabetes and obesity [17, 18]. Nevertheless, it is regrettable that there are few therapeutically effective and safe drugs for treating difficult-to-heal wounds, imposing severe economic and physiological burdens on society as well as patients.

Royal jelly, a traditional remedy for many kinds of cutaneous injuries, may have potential applications in the therapy of abnormal wounds. Limited previous studies have revealed that royal jelly, or its derivatives, have the effectiveness of promoting wound repair at both animal and cellular levels [10–12, 19–24], yet the subjacent mechanisms of action are still unclear. Besides, it is well-known that the compositions and bioactivities of royal jelly change with the honeybee species, environmental conditions, harvesting time and botanical sources [25]; however, there is no study investigating the wound-healing properties of royal jelly harvested in blossom seasons of different nectariferous plants, which may have hindered the application of specific royal jelly and the discovery of wound healing-promoting substances.

Here, this is the first study to compare the wound-healing activities of royal jelly collected during flowering seasons of *Castanea mollissima* Bl. (chestnut) and *Brassica napus* L. (rapeseed) by way of in vivo and in vitro wound healing assays. In the in vivo assay, excisional full-thickness wounds were created to preliminarily investigate the efficacy of different royal jelly in topical application. In the in vitro assays, human immortalised keratinocytes (HaCaT), resembling the normal keratinocytes making up the majority (95%) of epidermis, were employed to explore the proliferative and migratory activities of royal jelly extracts in wound healing. Moreover, anti-inflammatory effects of the extracts were evaluated in LPS-stimulated murine macrophages (RAW 264.7). This study will boost the development and application of particular royal jelly in wound healing, as well as the identification of biologically-active ingredients from royal jelly for use in different skin injury situations.

## Methods

### Collection of royal jelly samples

To ensure the relative purity and authenticity of botanical sources, raw royal jelly, produced by *Apis mellifera* L., was collected in the mid-term of flowering seasons of *Castanea mollissima* Bl. (chestnut) and *Brassica napus* L. (rapeseed), respectively, from apiaries in Zhejiang Province (29°50′N, 150°90′E), P.R. China, and was named CmRJ-Zj and BnRJ-Zj. Immediately after harvesting, samples were preserved at − 20 °C prior to use. No permission was necessary for the collection of royal jelly. *Castanea mollissima* Bl. and *Brassica napus* L. were authenticated by Yan Lin and the voucher specimens were deposited in the herbarium in the College of Animal Sciences (College of Bee Science), Fujian Agriculture and Forestry University, with deposition numbers of CmZj-20180601 and BnZj-20190401.

### In vivo wound healing assay

To preliminarily evaluate the differences in wound-healing activities between the raw royal jelly collected in flowering seasons of different floral sources, the in vivo wound healing assay was performed with samples CmRJ-Zj and BnRJ-Zj employing full-thickness wound models in rats. The experiments were performed according to the ARRIVE (Animals in Research: Reporting In Vivo Experiments) guidelines [26] and the Guidelines for the Care and Use of Medical Laboratory Animals (Ministry of Health, China, 1998). The procedure was approved and overseen by the Animal Care and Use Ethics Committee of Fujian Agriculture and Forestry University (No. PZCASFAFU2019008).

Adult female Wistar rats, approximately 12 weeks old, weight 180–250 g, were obtained from Animal Research Centre of Hubei Province. They were caged in a special room with a temperature of 25 ± 1 °C and a 12:12 light-dark cycle, and were supplied with a commercial pellet diet and water ad libitum. Before the experiment, the rats were maintained for 7 days to acclimatise to the laboratory environment. Five rats were randomly selected and anaesthetised by inhalation with 2% isoflurane in 98% air (surgical duration exposure) (RWD, Dover, USA) prior to shaving the dorsal skin hair and creating four full-thickness excision wounds (1.5-cm-diameter) on the back of each rat using biopsy punch. After surgery and clearance of the blood on the wounds, the images were photographed with a digital camera (Canon, Tokyo, Japan) and recorded as day 0. The four wounds in each rat were treated with 200 μl of solvent sterile saline (negative control), epidermal growth factor (EGF, 10 μg/ml, positive control), *Castanea mollissima* Bl. and *Brassica napus* L. royal jelly samples (10%) respectively every other day via topical administration and were left uncovered after application. Royal jelly and EGF were dissolved in sterile saline. Five rat replicates were included in the experiment. Wound areas were monitored on days 0, 2, 4, 6, 8, 10, 12 and 14, and photos were taken before each treatment. The open areas of wounds were calculated using Photoshop software (Adobe, San Jose, USA). After the study, rats were killed by anesthetisation with 2% pentobarbital sodium (0.1 ml/20 g body weight, i.p.) followed by cervical dislocation, in accordance with the Guidelines for the Care and Use of Medical Laboratory Animals. The researchers who were treating the wounds with agents and performing statistical analysis, were blinded. The area healed per day, between two adjacent observation time points (2 days), was calculated using the following formula:

$$ Area\ healed\  per\  day\ \left({cm}^2/ day\right)=\left({Area}_{n- 2}-{Area}_n\right)/\left({Day}_n-{Day}_{\mathrm{n}-2}\right)\times 100\% $$

The “Area_n-2_” and “Area_n_” were the areas of wounds on day n-2 and day n; the “Day_n_” and “Day_n-2_” were the day n and day n-2 post-wounding. As wound healing is usually delayed, and it is a nonlinear dynamic process, expressing the wound healing rate as a percent change in the area would be inaccurate when applied to wounds with varying initial sizes [27, 28]. To accurately reflect the dynamic changes in the wound healing extent over the time course and in order to eliminate the deviation caused by different initial wound sizes, here the wound healing was correlated with time to reflect the speed of wound closure within two contiguous observation time points, instead of merely comparing the wound sizes with the initial ones. The formula proposed above may potentially predict the time that it takes to complete wound healing, which is more meaningful for evaluating the efficacy of a treatment, as the goal of wound care is to shorten the wound closure process [27, 28].

### Cell culture

The immortalised human epidermal keratinocyte cell line (HaCaT cells, DSMZ No. 771) possessing basic characteristics of normal epidermal keratinocytes was purchased from DSMZ, Germany, and the murine macrophage cell line (RAW264.7 cells, No. TCM13) was obtained from the Cell Repository of the Chinese Academy of Sciences (Shanghai, China). They were cultured in Dulbecco’s Modified Eagle’s Medium (DMEM) (Hyclone, Logan, USA) with 10% foetal bovine serum (FBS) (Hyclone, Logan, USA) and 1% penicillin-streptomycin solution (TransGen, Beijing, China), and kept in a humidified incubator with 5% CO_2_ at 37 °C.

### Preparation of royal jelly extracts

Hydrophilic and lipophilic components of royal jelly were extracted similarly to Gismondi’s work [29]. In brief, 2.5 g of each royal jelly sample was suspended in 10 ml of PBS (Sangon, Shanghai, China), followed by vortexing for 30 min and centrifugation at 4 °C, 14,500×g for 10 min. The supernatants, hydrophilic extracts, were transferred into new centrifuge tubes and named CmRJ-Zj-HE and BnRJ-Zj-HE, respectively. The precipitation was resuspended in appropriate volume (0.3125–10 ml) of DMSO, ensuring the final concentration of DMSO present in the experimental groups in the subsequent bioassays was lower than 0.1% to avoid cytotoxicity, and then the suspension was centrifuged again in the same way as before. The resultant supernatants were lipophilic extracts and named CmRJ-Zj-LE and BnRJ-Zj-LE, respectively. Both the hydrosoluble and the liposoluble extracts were preserved as stock solutions (250 mg/ml or 8 g/ml) at − 20 °C prior to use, while the remaining pellets were discarded.

### MTT cell viability assay

The proliferative and cytotoxic effects of royal jelly extracts on HaCaT and RAW 264.7 cells were evaluated with MTT colorimetric assay. It was conducted as described in our previous work with minor modification [21]. Briefly, 100 μl of HaCaT and RAW 264.7 cell suspension in DMEM at a density of 3.5 × 10^4^ and 1 × 10^7^ cells/ml, respectively, was added to each well of 96-well plates, followed by 24-h incubation. Royal jelly extracts were diluted from the stock solution (250 mg/ml) with serum-free DMEM. Before being treated with a series of extracts at different concentrations for 48 h (HaCaT cells) or 24 h (RAW 264.7 cells), cells were starved for 12 h in serum-free DMEM. Control cells were treated with serum-free DMEM containing the highest amount of PBS or DMSO present in test groups (0.1%). Then, 10 μl of 3-(4,5-Dimethylthiazol-2-yl)-2,5-diphenyltetrazolium bromide (MTT, 5 mg/ml, Beyotime, Shanghai, China) was added for reaction for about 4 h. After removal of the supernatants, formazan crystals were dissolved in 100 μl of DMSO, and the absorbance was measured at 492 nm in an Infinite F50 plate reader (Tecan, Männedorf, Austria). Tests were repeated at least three times for each extract sample. Cell viability was calculated using the formula below:

$$ Cell\ viability\ \left(\%\right)={A}_x/{A}_0\times 100\% $$

The “A_x_” was the absorbance of royal jelly extracts treated cells; the “A_0_” was the absorbance of serum-free medium treated cells.

### In vitro scratch wound assay

The migratory effects of extract samples on HaCaT cells were assessed by scratch wound assay. Scratch wounds (500-μm-wide) were created by placing Culture-Inserts (ibidi, Martinsried, Germany) into each well of 24-well plates before culturing 70 μl of HaCaT cells (5 × 10^5^ cells/ml) in each cell of the Inserts for around 18 h to form confluent cell monolayers. Culture-Inserts were removed discreetly and detached cells were washed off with PBS (Sangon, Shanghai, China). Royal jelly extracts were diluted from the stock solution (250 mg/ml) with serum-free DMEM. Cells were then treated with 2 ml of sample solutions at specified concentrations for 24 h. Control cells were treated with serum-free DMEM containing the highest amount of PBS or DMSO present in test groups (0.0125%). The scratch wounds were observed under an inverted microscope (Olympus, Tokyo, Japan). Images of three, randomly selected, cell-free regions in each well were captured at 0, 12 and 24 h following wounding, and analysed using Image J software (National Institutes of Health, Bethesda, MD, USA). Three replicates were set for each concentration and three independent experiments were conducted. Wound closure rates were calculated using the following formula:

$$ Wound\ closure\ rate\ \left(\%\right)=\left({Area}_0-{Area}_{12/24}\right)/{Area}_0\times 100\% $$

The “Area_0_” was the scarification area at 0 h; the “Area_12/24_” was the scarification area at 12 or 24 h, respectively.

### Determination of anti-NO production activity

The production of nitric oxide (NO) by RAW 264.7 cells was determined based on the nitrite present in the culture supernatants using Griess reagent (Congyi, Shanghai, China). RAW 264.7 cells (5.0 × 10^6^ cells/well) were seeded into 24-well plates. After 12-h incubation, medium was replaced with serum-free DMEM with or without royal jelly extracts for a further 1-h incubation. Royal jelly extracts were diluted from the stock solution (8 g/ml) with serum-free DMEM. The final concentrations of DMSO present in control and experimental groups were lower than 0.05%. LPS from *Escherichia coli* (Sigma-Aldrich, Saint Louis, USA) was added to a final concentration of 1 μg/ml [30, 31]. About 24 h later, the culture supernatants were collected and reacted with 100 μl of Griess reagent composed of 1% sulfanilamide in 5% phosphoric acid and 0.1% N-(1-naphthyl) ethylenediamine for 10 min at room temperature in a dark place. The optical density was measured at 540 nm in an Infinite F50 plate reader (Tecan, Männedorf, Austria). Three independent experiments with four replicates in each concentration were conducted. NO production was presented as:

$$ \% of\  LPS={A}_{\mathrm{x}}/{A}_0\times 100\% $$

The “A_x_” was the absorbance of cells exposed to royal jelly extracts and LPS; the “A_0_” was the absorbance of cells exposed to LPS alone.

### Enzyme-linked immunosorbent assay (ELISA)

To measure the secretion of cytokines modulated by royal jelly extracts in RAW 264.7 cells, the culture supernatants collected above for the measurement of NO production was also tested for the generation of TNF-α (tumour necrosis factor-α) and TGF-β1 (transforming growth factor-β1) using ELISA kits (Dakewe, Beijing, China). Briefly, 5.0 × 10^6^ RAW 264.7 cells were seeded into each well of 24-well plates and incubated for 12 h. Cells were treated with serum-free DMEM with or without royal jelly extracts for 1 h before the treatment of LPS (1 μg/ml) for a further 24 h [30, 31]. The final concentrations of DMSO present in control and experimental groups were lower than 0.05%. Cell supernatants were collected for the detection of TNF-α and TGF-β1 according to the manufacturer’s instructions. Three independent experiments with four replicates in each concentration were conducted. TNF-α or TGF-β1 production was presented as:

$$ \% of\  LPS={A}_{\mathrm{x}}/{A}_0\times 100\% $$

The “A_x_” was the absorbance of cells exposed to royal jelly extracts and LPS; the “A_0_” was the absorbance of cells exposed to LPS alone.

### Statistical analysis

Data were processed using GraphPad Prism 5.0 software (GraphPad Software Inc., CA, USA). Comparisons of variance were performed using one-way ANOVA, in which *p* < 0.05 was considered as statistically significant. Results were expressed as means ± SEM.

## Results

### Wound-healing activity of royal jelly on full-thickness excision skin of rats

**Fig. 1:**
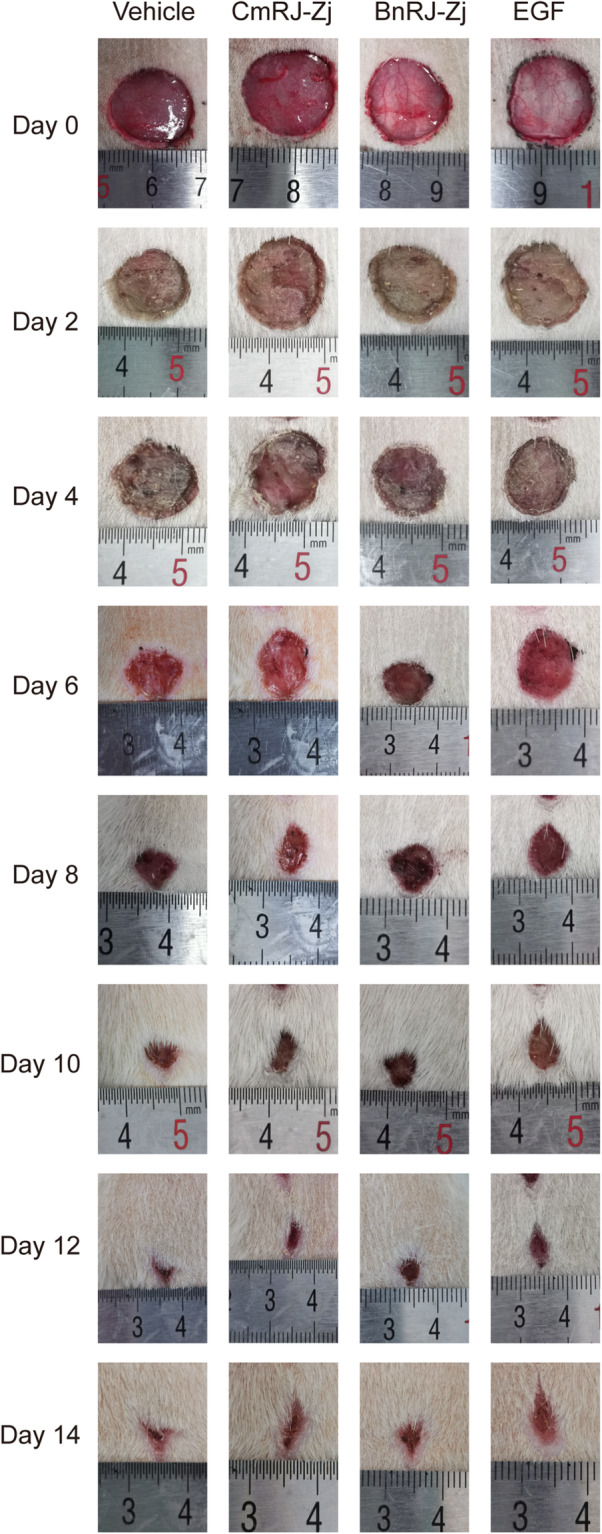
Effects of topical application of royal jelly on full-thickness wounds in rats. Photographs of wounds from a representative rat captured on post-wounding days 0, 2, 4, 6, 8, 10, 12 and 14. Wounds were treated with 200 μl of sterile saline (negative control), epidermal growth factor (EGF, 10 μg/ml, positive control), 10% *Castanea mollissima* Bl. royal jelly (CmRJ-Zj) or 10% *Brassica napus* L. royal jelly (BnRJ-Zj) every other day

In the skin excisional rat models, after treatment, all the rats (5/5) were healthy and no microbial infection was observed. As shown in Table 1 and Fig. 1, on day 2, the open wounds treated with *Castanea mollissima* Bl. royal jelly (CmRJ-Zj) and the epidermal growth factor (EGF) were larger than the initial sizes, and the corresponding areas healed per day in the initial 2 days were significantly lower than the control group. Surprisingly, in spite of the graver wounds after CmRJ-Zj treatment in the first 2 days, the areas healed per day of these wounds between day 2 and day 4 were statistically higher than the control (*p* < 0.05) (Table 1). Nevertheless, since the average areas and the areas healed per day of the wounds treated with EGF throughout the time course had no statistical difference with those treated with vehicle (Table 1), it seemed that EGF did not exhibit in vivo wound healing-promoting activity in this study. Similarly, *Brassica napus* L. royal jelly did not improve the wound repair at all. As royal jelly is mainly composed of proteins which may be degraded in the complicated wound environment, resulting in abolishment of the bioactivity, it would be worthwhile performing a series of in vitro assays to further assess the potential wound-healing function of royal jelly.

**Table 1:** Wound areas and healing speed at each observation time point during the procedure of treatment

Groups	Average of wound areas (cm^**2**^) (mean ± SEM)								Area healed per day (cm^**2**^/day) (mean ± SEM)						
	Day 0	Day 2	Day 4	Day 6	Day 8	Day 10	Day 12	Day 14	Day 2	Day 4	Day 6	Day 8	Day 10	Day 12	Day 14
**Solvent sterile saline (negative control)**	1.802 ± 0.021	1.406 ± 0.024	1.144 ± 0.023	0.618 ± 0.030	0.308 ± 0.014	0.170 ± 0.023	0.090 ± 0.015	0.036 ± 0.008	0.198 ± 0.016	0.131 ± 0.017	0.263 ± 0.012	0.155 ± 0.015	0.069 ± 0.006	0.040 ± 0.004	0.027 ± 0.004
**EGF (10 μg/ml, positive control)**	1.760 ± 0.020	1.796 ± 0.014***	1.388 ± 0.022**	0.862 ± 0.016*	0.422 ± 0.018	0.286 ± 0.012	0.146 ± 0.011	0.048 ± 0.009	−0.018 ± 0.010^##^	0.204 ± 0.014	0.263 ± 0.012	0.220 ± 0.004	0.068 ± 0.008	0.070 ± 0.004	0.049 ± 0.003
***Castanea mollissima*** **Bl. royal jelly (10%)**	1.782 ± 0.022	1.926 ± 0.007***	1.426 ± 0.016**	0.764 ± 0.027	0.450 ± 0.022	0.284 ± 0.015	0.136 ± 0.013	0.046 ± 0.010	−0.072 ± 0.013^###^	0.250 ± 0.007*	0.331 ± 0.013	0.157 ± 0.007	0.083 ± 0.006	0.074 ± 0.006	0.045 ± 0.003
***Brassica napus*** **L. royal jelly (10%)**	1.768 ± 0.019	1.334 ± 0.027	1.080 ± 0.012	0.716 ± 0.027	0.338 ± 0.016	0.182 ± 0.010	0.084 ± 0.005	0.036 ± 0.005	0.217 ± 0.020	0.127 ± 0.012	0.182 ± 0.009	0.189 ± 0.006	0.078 ± 0.004	0.049 ± 0.004	0.024 ± 0.001

Area healed per day (cm^2^/day) = (Area_n-2_-Area_n_) / (Day_n_-Day_n-2_) × 100%. The “Area_n-2_” and “Area_n_” were the areas of wounds on day n-2 and day n; the “Day_n_” and “Day_n-2_” were the day n and day n-2 post-wounding. Statistical significance of differences: *, *p* < 0.05; **, *p* < 0.01; ***, *p* < 0.001; ##, *p* < 0.01; ###, *p* < 0.001 (* and # represent the values are higher and lower than the negative control, respectively)

### Proliferative effects of royal jelly extracts on human epidermal keratinocytes

**Fig. 2:**
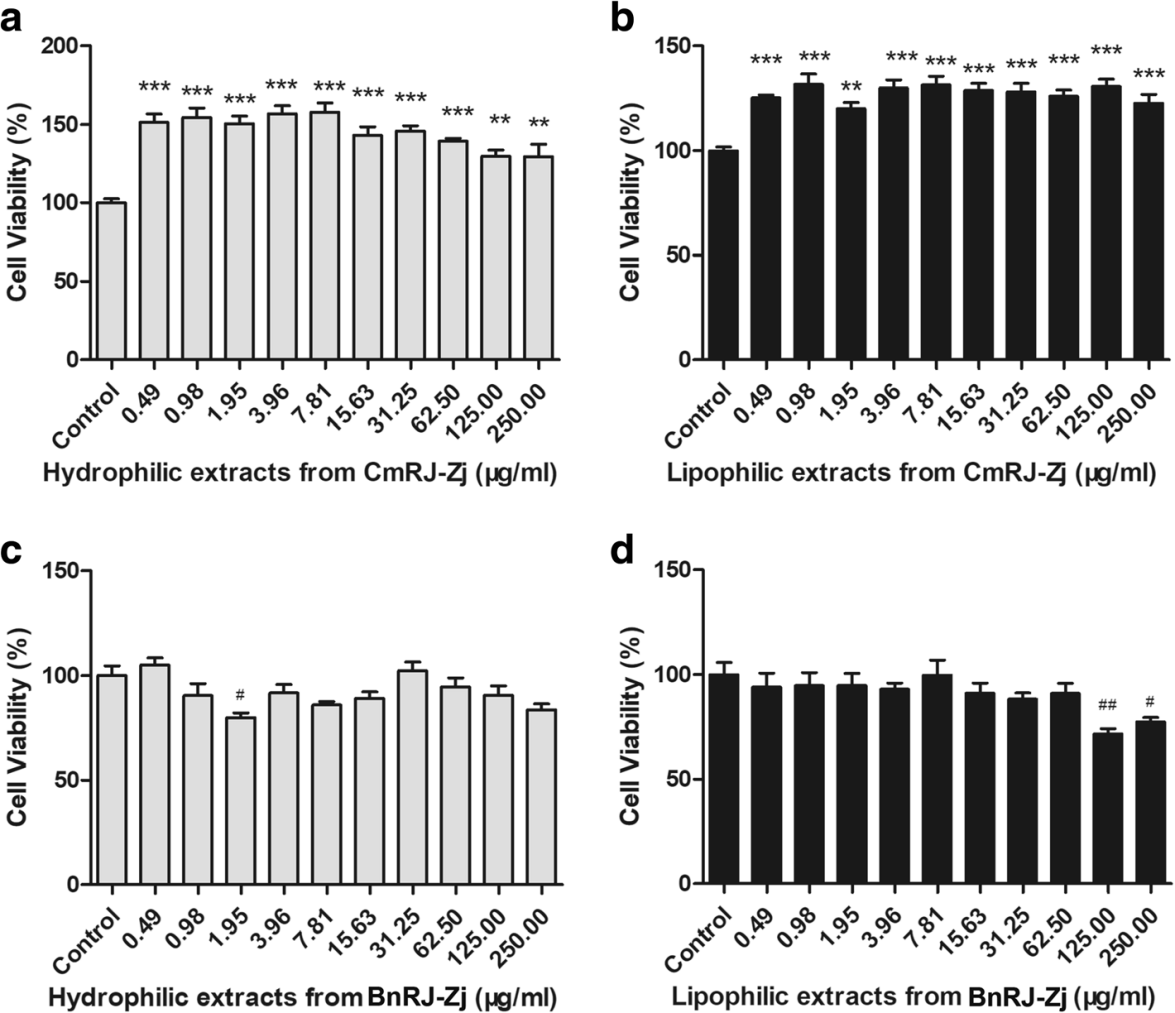
Proliferative effects of royal jelly extracts on keratinocytes (HaCaT) after incubation for 48 h. Effects of hydrophilic and lipophilic fractions from *Castanea mollissima* Bl. **a**-**b** and *Brassica napus* L. **c**-**d** royal jelly on the growth of HaCaT cells. Results are expressed as the mean ± SEM. Statistical significance of differences: **, *p* < 0.01; ***, *p* < 0.001; #, *p* < 0.05; ##, *p* < 0.01 (* and # represent proliferative and cytotoxic effects compared with control conditions, respectively)

The preliminary in vivo wound healing study indicated that royal jelly harvested in the flowering seasons of *Castanea mollissima* Bl. and *Brassica napus* L. exhibited remarkably different wound healing activities. As the compositions and bioactivities of royal jelly may be affected by botanical sources, the hydrosoluble and liposoluble components were extracted from both royal jelly to further investigate the influence of different chemical components on their medical functions. As shown in Fig. 2, both hydrophilic and lipophilic fractions from *Castanea mollissima* Bl. royal jelly significantly promoted the proliferation of HaCaT cells without any cytotoxicity; in contrast, fractions from *Brassica napus* L. royal jelly could not enhance cell growth at the concentrations up to 250.00 μg/ml and might even cause cell death at some doses. It suggested that *Castanea mollissima* Bl. royal jelly might facilitate wound repair via its cell-growth promoting efficacy, while *Brassica napus* L. royal jelly might lack such potential.

### Migratory effects of royal jelly extracts on the in vitro scratched wounds

**Fig. 3:**
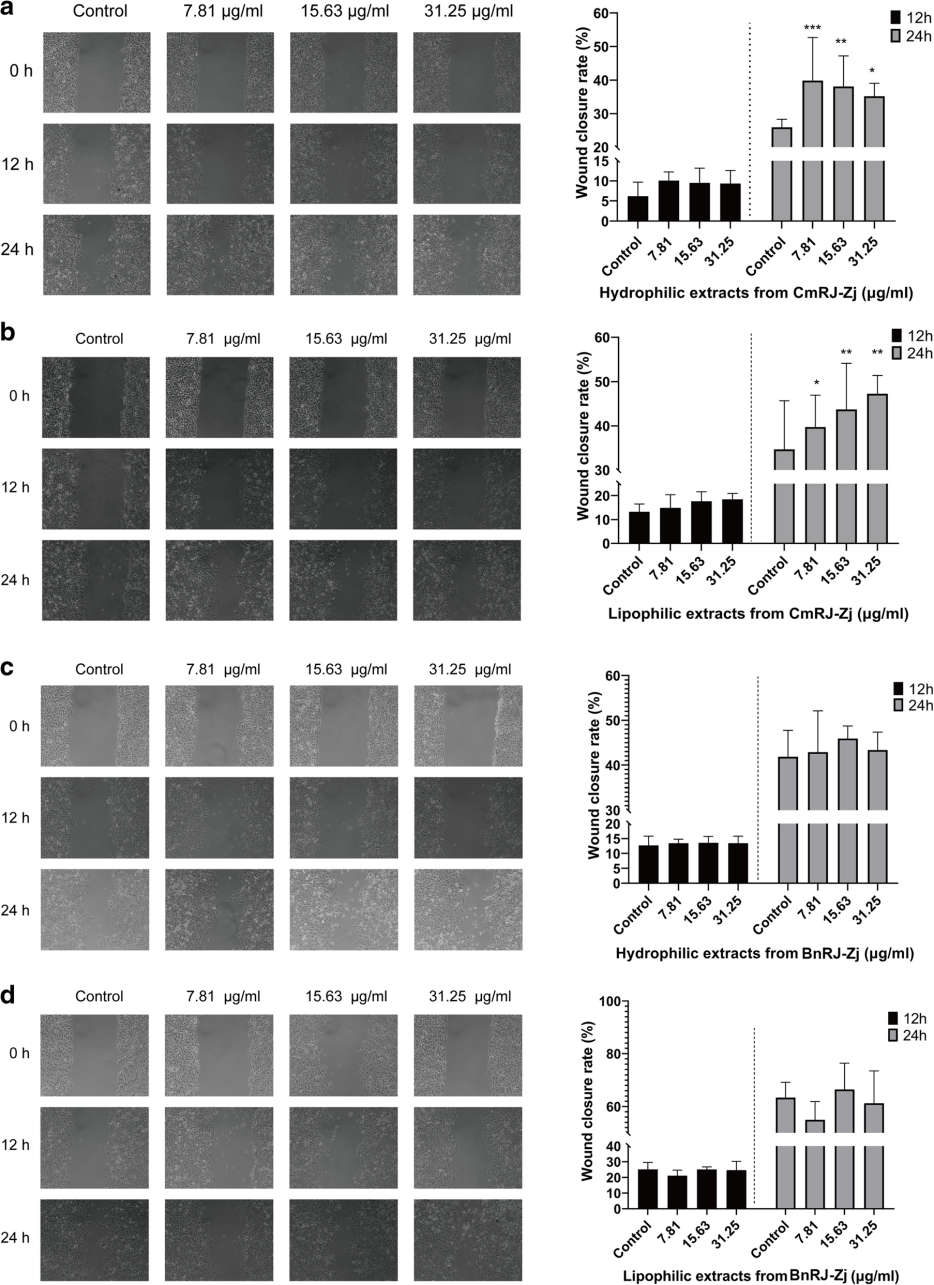
Migratory effects of royal jelly extracts on the in vitro keratinocytes scratched wounds. Effects of hydrophilic and lipophilic extracts from *Castanea mollissima* Bl. **a**-**b** and *Brassica napus* L. **c**-**d** royal jelly on HaCaT cell migration. The left panels are micrographs of cell migration observed at × 100 magnification. The right panels are rates of wound healing calculated from the area of wound coverage over a period of 12 or 24 h relative to the scarification area at 0 h. Results are expressed as the mean ± SEM. Statistical significance of differences: *, *p* < 0.05; **, *p* < 0.01; ***, *p* < 0.001, compared with control conditions

The extracts from *Castanea mollissima* Bl. and *Brassica napus* L. royal jelly were further investigated for the effects on cell migration. It is obvious that, at 24 h post-wounding, both hydrophilic and lipophilic extracts from *Castanea mollissima* Bl. royal jelly dramatically boosted the wound closure of human keratinocytes in vitro compared with control (Fig. 3a-b). More specifically, hydrophilic extract of *Castanea mollissima* Bl. royal jelly showed the highest wound healing rate (40%) at 7.81 μg/ml (Fig. 3a), and lipophilic extract exhibited dose-dependent re-epithelialisation effects (Fig. 3b). On the other hand, no distinct enhancement in cell migration could be observed in the scratched wounds treated with either hydrosoluble or liposoluble extracts from *Brassica napus* L. royal jelly at tested concentrations after 12- or 24-h incubation in comparison with control (Fig. 3c-d). Hence, the wound-healing activity of *Castanea mollissima* Bl. royal jelly may also be attributed to its stimulant effects on the migration of keratinocytes.

### Anti-inflammatory effects of royal jelly extracts on LPS-stimulated murine macrophages

**Fig. 4:**
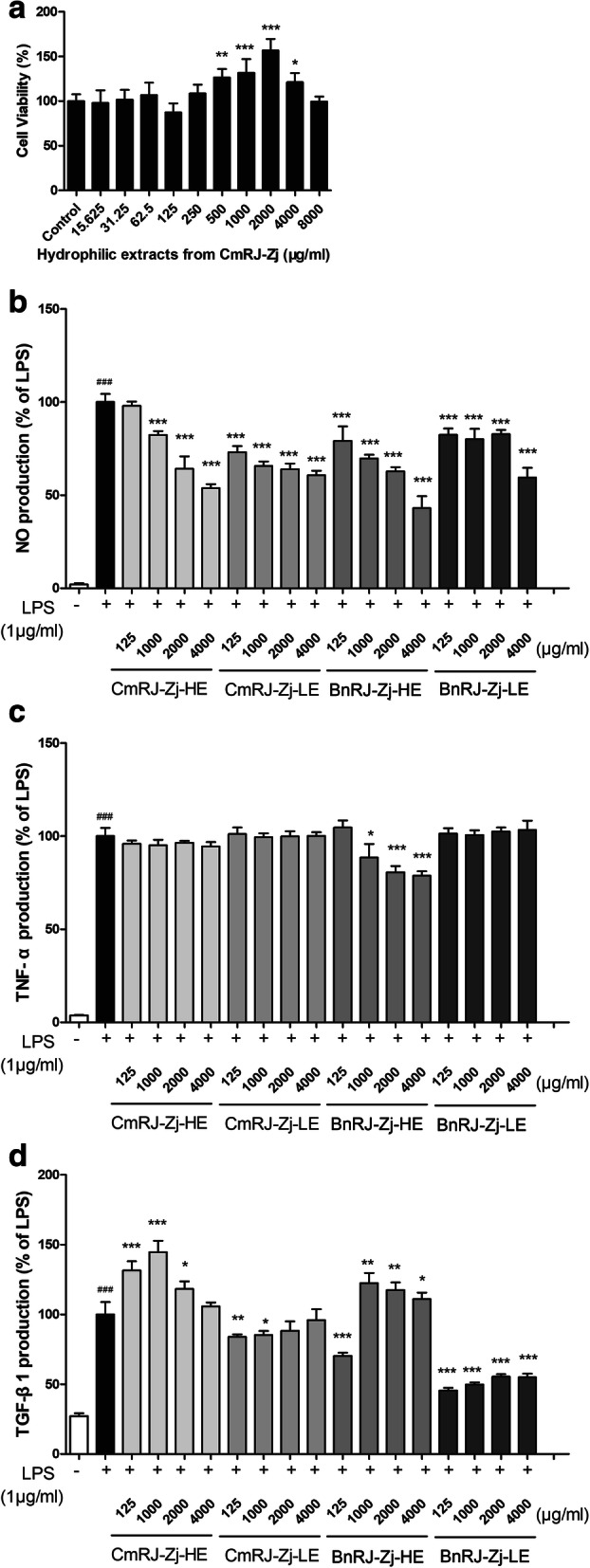
Effects of royal jelly extracts on the cell viability and LPS-induced mediators production in murine macrophages. **a** Cytotoxic effects of hydrophilic extracts from *Castanea mollissima* Bl. royal jelly on RAW 264.7 cells as determined by MTT assay. *, *p* < 0.05; **, *p* < 0.01; ***, *p* < 0.001, compared with control. Effects of royal jelly extracts on the production of NO (**b**), TNF-α (**c**) and TGF-β1 (**d**) in LPS-induced RAW 264.7 cells. CmRJ-Zj-HE, hydrophilic extracts from *Castanea mollissima* Bl. royal jelly; CmRJ-Zj-LE, lipophilic extracts from *Castanea mollissima* Bl. royal jelly; BnRJ-Zj-HE, hydrophilic extracts from *Brassica napus* L. royal jelly; BnRJ-Zj-LE, lipophilic extracts from *Brassica napus* L. royal jelly. Results are expressed as the mean ± SEM. ###, *p* < 0.001, compared with control (vehicle treated cells). *, *p* < 0.05; **, *p* < 0.01; ***, *p* < 0.001, compared with LPS-stimulated cells

Before exploring the anti-inflammatory effects of royal jelly extracts on macrophages, the cytotoxicity towards the cells was examined via MTT cell viability assay. None of the extracts from royal jelly affected the viability of RAW 264.7 cells at concentrations lower than 4000 μg/ml. Take hydrophilic extracts from *Castanea mollissima* Bl. royal jelly for example, it was found to be devoid of toxicity towards RAW 264.7 cells in the range of 15.63–4000 μg/ml, in which it even significantly promoted the growth of macrophages at the concentrations from 500 to 4000 μg/ml (Fig. 4a). Hence, concentrations below 4000 μg/ml were used for the subsequent investigation of the anti-inflammatory effects.

Regarding the in vitro inflammation in macrophages, LPS significantly induced the production of NO and TNF-α, whereas it was improved by the treatment of either hydrosoluble or liposoluble extracts from royal jelly samples to various extents. More specifically, exposed to various extracts, NO production in RAW 264.7 cells was dramatically reduced at almost all the tested concentrations in a dose-dependent manner, in which the effects of hydrophilic extracts from both *Castanea mollissima* Bl. and *Brassica napus* L. royal jelly were slightly better than those of lipophilic extracts (Fig. 4b). In terms of the TNF-α production in LPS-induced RAW 264.7 cells, except for the marked suppression caused by the hydrophilic extracts from *Brassica napus* L. royal jelly, none of the other samples showed anti-inflammatory potential (Fig. 4c). Interestingly, the hydrophilic and lipophilic extracts from royal jelly exhibited completely different effects on the production of TGF-β1 in LPS-stimulated RAW 264.7 cells. The amount of TGF-β1 was significantly increased by the treatment of hydrosoluble extracts from both *Castanea mollissima* Bl. and *Brassica napus* L. royal jelly, and the enhancement caused by *Castanea mollissima* Bl. royal jelly was superior to *Brassica napus* L. royal jelly (Fig. 4d). Conversely, liposoluble extracts from both royal jelly remarkably reduced the secretion of TGF-β1 which was decreased to about 50% by the lipophilic extracts from *Brassica napus* L. royal jelly with respect to the LPS-stimulated cells (Fig. 4d). The data suggested that extracts from *Castanea mollissima* Bl. and *Brassica napus* L. royal jelly exerted anti-inflammatory or growth-motivating activities to different extents through mediation of cytokines production in LPS-stimulated macrophages, implying that different components had distinct mechanisms for wound healing.

## Discussion

Chronic wounds refer to ones that cannot produce anatomically and functionally integrated tissues within 3 months, in a timely and orderly manner [32]. It has been a severe problem in clinic, as non-healing wounds impose physiological and financial burdens on patients and society throughout the world. Approximately 1–2% of the population in developed countries is predicted to suffer from cutaneous chronic wounds in the future [32], and unfortunately, almost no therapeutically effective and safe drug is available for treating the wounds, which aggravates the problem more seriously. Since ancient times, royal jelly, produced by nurse bees, has been used as a supplementary therapy for the treatment of various skin injuries [19]. Nevertheless, the modern application of royal jelly in skin disorders is rarely reported, which may be attributed to the lack of knowledge about the wound-healing mechanisms or the diversity and uncertainty of the components. Thus, this study preliminarily compared the in vivo wound-healing activities of royal jelly harvested in the blossom seasons of *Castanea mollissima* Bl. and *Brassica napus* L., as well as the in vitro modulatory effects of their extracts on wound repair.

In the in vivo wound healing assay, excisional full-thickness wounds were created on the dorsal skin of Wistar rats. When evaluating the wound healing-promoting activity, −unlike many other studies measuring the wound healing with absolute changes of open wound areas, or with percentages of healed wounds relative to the initial areas [19, 27, 28], −we estimated the effects of royal jelly by calculating the healing speed (area healed per day, cm^2^/day) between two adjacent observation time points [27]. Wound healing is a nonlinear process, yet the commonly used calculation neglects the nature of dynamic wound healing and differences in the initial wound sizes, so that a greater change in area may be generated from the relatively small amount of healing in a larger wound than in a smaller wound [27, 28]. In contrast, the formula used in this study could eliminate such misleading results to some extent [27, 28]. As the statistical analysis in Table 1, the wounds treated with *Castanea mollissima* Bl. royal jelly enlarged on 2 days post-wounding, thus it further confirmed that traditional formulae could not reflect the detailed wound healing process, and instead, the wound healing should be evaluated separately at each time point. It was illustrated in Table 1 that the wound area healed per day in the initial 2 days in *Castanea mollissima* Bl. royal jelly group was significantly lower than the control group (*p* < 0.001), whereas between day 2 and day 4 (calculated on day 4) it was shown to be statistically higher than the control (*p* < 0.05). We speculated that the larger wounds on day 2 might be in the inflammatory stage of wound healing, in which no obvious contraction and scarring could be observed, as phagocytic cells were not responsible for filling in the defects [16], and *Castanea mollissima* Bl. royal jelly might not have any potency on the early inflammation. The higher healing speed on the wounds exposed to *Castanea mollissima* Bl. royal jelly between day 2 and day 4 might be attributed to its anti-inflammatory effects on macrophages that usually infiltrated the wound sites at around 48 h after injury [16]; the weak potency of royal jelly might also be owing to the degradation of royal jelly since its water-soluble proteins were reported to evoke potential wound healing effects [21]. In the subsequent assays with LPS-stimulated macrophages, the anti-inflammatory efficacy of *Castanea mollissima* Bl. royal jelly was confirmed (Fig. 4b). Surprisingly, epidermal growth factor (EGF) did not promote wound closure in the current study, which might be caused by the instability of EGF or the low frequency of treatment [33]. According to the study conducted by El-Gayar et al. [11], 50 and 100% of royal jelly administered twice daily improved wound closure in rat infected excisional wound models, while fresh royal jelly (10%) or EGF applied every other day in this study did not display marked wound healing improvement in non-infected wounds. Accordingly, in the future, the doses and dosing frequency may need to be increased to reduce the undesired impacts caused by the degradation of royal jelly proteins and EGF.

Since *Castanea mollissima* Bl. and *Brassica napus* L. royal jelly exhibited slightly different wound healing effectiveness, the subjacent mechanisms of action were investigated with in vitro assays. It was found that both hydrophilic and lipophilic extracts from *Castanea mollissima* Bl. royal jelly could promote the proliferation and migration of keratinocytes, while both extracts from *Brassica napus* L. royal jelly were devoid of such effects (Figs. 2 and 3), implying that *Castanea mollissima* Bl. royal jelly might accelerate wound healing via facilitating re-epithelialisation.

In terms of anti-inflammatory effects, it is well-known that inflammation takes place immediately after skin injury, protecting wounds from infection and facilitating subsequent proliferation; however, prolonged inflammation may lead to delayed healing [14, 16, 34]. Hence, substances with anti-inflammatory activity are considered to have wound healing-promoting potential. In this study, extracts from both *Castanea mollissima* Bl. and *Brassica napus* L. royal jelly supressed the production of NO in LPS-induced RAW 264.7, and those from *Castanea mollissima* Bl. royal jelly were more potent (Fig. 4b). LPS is a stimulus that can induce the secretion of pro-inflammatory cytokines and mediators, including NO and TNF-α [35, 36]. NO is generated by the catalysis of NO synthase (iNOS) and it influences cell growth, mobility, differentiation, and inflammatory response [13, 37]. It promotes cell proliferation and migration at low concentration (< 50 nM), while exhibiting the opposite effects at high concentration (> 100 nM) [13, 38]. Thus, the inhibitory activities of royal jelly extracts on the production of NO (more than 50 μM was generated by LPS-stimulation in this study) were beneficial to shorten inflammation and enhance re-epithelialisation in the wound healing process. In the meantime, the hydrophilic extracts from *Brassica napus* L. royal jelly could also repress LPS-stimulated production of TNF-α which was a kind of crucial pro-inflammatory cytokines promoting wound healing via inducing inflammation yet being surplus at chronic wound sites [39] (Fig. 4c). Thus, the hydrophilic extracts from *Brassica napus* L. royal jelly might regulate wound healing through controlling inflammation. Besides, hydrophilic extracts from both royal jelly could enhance the production of TGF-β1, while the lipophilic extracts displayed converse effects (Fig. 4d). TGF-β1 is a growth factor motivating the proliferation of cells involved in wound healing. Thus, the hydrophilic extracts from royal jelly might also boost wound closure by increasing the secretion of growth factors.

Based on the in vivo and in vitro studies, we found that *Castanea mollissima* Bl. royal jelly possessed more potent wound-healing potential, through enhancing keratinocytes growth and migration, reducing NO production and increasing TGF-β1 generation. These modulatory effects might contribute to the wound healing potency observed in the animal assays in this study. Furthermore, it was also found that the anti-inflammatory efficacies of hydrophilic extracts of each royal jelly were stronger than those of lipophilic extracts. We speculate that it may be possible to treat non-healing wounds at different stages with different components from specific royal jelly and the bioactive substances are more likely to be water-soluble. In the future, it will be worthwhile to analyse the chemical components present in *Castanea mollissima* Bl. and *Brassica napus* L. royal jelly to verify the exact compounds evoking wound healing-promoting efficacy.

This is the first study to compare the wound-healing potencies of royal jelly derived from blossom seasons of different nectar plants, and to preliminarily investigate the underlying mechanisms of their hydrophilic and lipophilic extracts, providing insights into the identification of bioactive compositions from particular royal jelly for the future of wound care. Nevertheless, there were several limitations to this study. The doses and dosing frequency administered to rats might have been too low to improve wound closure. The wounds treated with *Castanea mollissima* Bl. royal jelly and EGF were larger than the initial ones on day 2, greatly interfering with the analysis on the therapeutic effects, thus a greater number of rats should be tested in the future. Also, following the expansion of wounds on day 2 in the *Castanea mollissima* Bl. royal jelly group, there was a statistical difference in the wound healing speed compared with control between day 2 and day 4, thus histological and immunohistochemical analyses might be worth carrying out to explore the pathological and physiological changes in this period.

## Conclusion

This study indicated that royal jelly, collected during blossom seasons of different nectariferous plants, modulated wound healing with distinct mechanisms. Over the past number of decades, chronic wounds have been a global healthcare issue, due to the aging population and the rise in the incidence of diabetes and obesity. Inspired by this study, some problematic wound models, such as diabetic foot ulcers, infected cutaneous wounds and even MRSA skin infections, could be established to explore the scope of application of specific royal jelly. This would facilitate the development of royal jelly for wound care, and the discovery of wound healing-promoting components to deal with skin injuries at different stages or with different symptoms.

## Data Availability

The datasets used and/or analysed during the current study available from the corresponding author on reasonable request.

## Abbreviations

DMEM: Dulbecco’s Modified Eagle’s Medium
EGF: Epidermal growth factor
FBS: Foetal bovine serum
HaCaT: Human epidermal keratinocytes
MTT: 3-(4, 5-Dimethylthiazol-2-yl)-2,5-diphenyltetrazolium bromide
NO: Nitric oxide
RAW 264.7: Murine microphages
TGF-β: Transforming growth factor-β
TNF-α: Tumour necrosis factor-α
